# Elevated Electrode Impedances During Deep Brain Stimulation Surgery May Be Due to Peri-Electrode Air Collections

**DOI:** 10.7759/cureus.21518

**Published:** 2022-01-23

**Authors:** Bryan T Klassen, Juliana Rotter, Colleen Crane, Timothy J Kaufmann, Kai J Miller

**Affiliations:** 1 Neurology, Mayo Clinic, Rochester, USA; 2 Neurological Surgery, Mayo Clinic, Rochester, USA; 3 Neuromodulation, Boston Scientific, Valencia, USA; 4 Neuroradiology, Mayo Clinic, Rochester, USA

**Keywords:** ct (computed tomography) imaging, computed tomography, impedance, segmented lead, directional stimulation, neurosurgery, deep brain stimulation

## Abstract

Deep brain stimulation (DBS) is a commonly used treatment for medically refractory movement disorders and epilepsy. Intraoperative testing of electrode impedances is routinely done during DBS surgery to identify electrical conduction defects in the system. We present two illustrative cases involving elevated intraoperative impedances. In the first case, the temporal evolution of impedance changes and a postoperative head CT were consistent with a small and slowly resolving air collection along the lead. In the second case, an abnormally high impedance reading was observed at a single electrode and then “transferred” to be observed at an adjacent electrode upon adjustments of the electrode position, likely due to small air collection at a fixed position in the brain tissue. In both cases, careful troubleshooting allowed identification of the issue and avoidance of unnecessary surgical revisions. A thorough understanding of the possible sources of, and troubleshooting for, abnormal impedance readings is needed for effective intraoperative DBS monitoring.

## Introduction

Deep brain stimulation (DBS) is a well-established therapy for movement disorders (including essential tremor, Parkinson's disease, and dystonia) and certain forms of epilepsy and may potentially become standard therapy for other neurological or psychiatric diseases including Tourette syndrome, obsessive-compulsive disorder, and depression. A prerequisite for effective DBS therapy is a properly functioning implanted DBS system; however, DBS hardware complications may occur in a minority of cases [1].

Testing the impedance to current flow in an implanted DBS system or its individual components is an essential tool to identify an open or short circuit. This is often done intraoperatively, immediately after DBS lead implantation, to ensure that the implanted lead has no electrical connectivity defects. However, factors external to the DBS system also affect the impedance; these include tissue properties at the electrode interface as well as the presence of air or fluid between the electrode surface and the target tissue.

In this report, we present two illustrative DBS cases in which elevated intraoperative electrode impedances were suggestive of hardware malfunction but were in fact due to small pockets of air approximating the electrode. We review practical troubleshooting steps one can take to identify the underlying etiology of abnormal impedance readings intraoperatively.

## Case presentation

Case 1

**Figure 1:**
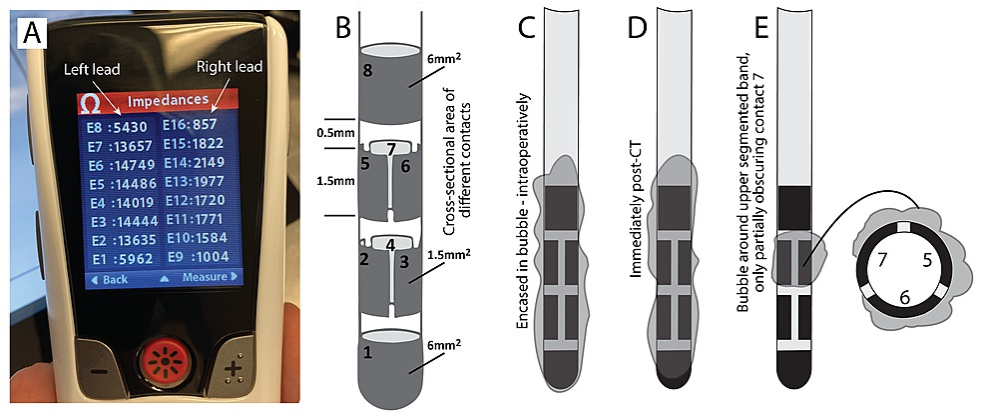
Example case 1 (A) Intraoperative impedance measurements from the patient remote, showing abnormally high values in the left lead compared to the right lead. (B) Schematic of implanted electrode segment geometry. (C-E) Cartoon simulations of the peri-lead air collection at the time of impedance testing intraoperatively (C), post-implantation head CT (D), and on postoperative day 1 as impedances began to improve (E).

A 60-year-old left-handed male with a history of medically refractory essential tremor was implanted with a bilateral Vim thalamic DBS system. The right Vim electrode was placed first, followed by the left. For each side, (1) a single-trajectory microelectrode recording confirmed the borders surrounding the Vim nucleus; (2) test stimulation applied through the reference ring of the microelectrode cannula elicited tremor improvement and only mild paresthesia bilaterally; (3) the permanently implantable directional stimulating lead (Boston Scientific Vercise Cartesia model, DB-2202-45) was placed; (4) the implanted lead was connected to a test cable (DB-4100A) that was plugged into an external test stimulator (DB-9315); (5) impedances were checked using a clinician programmer (DB-7161) and the external test stimulator and found to be normal on all of the lead’s electrodes; (6) test stimulation using the distal electrode as cathode and proximal as anode was found to significantly improve tremor with only mild sensory side-effects at higher currents; (7) a strain relief loop was positioned and the lead was secured using a standard burr hole cover/lead fixation device (SureTek, DB-4600-C); (8) impedance testing using the external test stimulator was repeated and again found to be normal (with an impedance ratio between circumferential and segmented electrodes of roughly 1:1.5) (Table 1, Column A; Figure 1); and (9) the proximal end of the lead was tucked under the scalp for later attachment to its lead extension.

**Table 1:** Electrode impedances (in ohms) on the lead illustrated in case 1 See Figure 1. IPG, Implantable pulse generator; ETS, external trial stimulator.

	A	B	C	D	E	F	G
	Post Lead Secured	Post IPG Connection	Post IPG Connection	Post Valsalva	Post CT	Post-op Day 1	Clinic 3 weeks
Stimulator	ETS	IPG	IPG	IPG	IPG	IPG	IPG
Programmer	Clinician	Clinician	Patient	Patient	Patient	Patient	Clinician
E8	1506	9999+	9810	5430	5778	883	877
E7	2686	9999+	13429	13657	13303	3088	1599
E6	2421	9999+	13854	14749	13835	17490	1569
E5	2584	9999+	14427	14486	13488	12715	1418
E4	2243	9999+	13989	14019	14111	1534	1803
E3	2543	9999+	14658	14444	14309	1896	2010
E2	2571	9999+	14021	13635	13467	1421	1790
E1	1791	9999+	10025	5962	2115	769	1238
Ratio	1.5	1	1.4	2.5	3.5	7.7	1.6

The patient was then placed under general anesthesia for tunneling of the lead extensions (NM-3138-55) and placement of the implantable pulse generator (IPG) (DB-1200) in a left infraclavicular pocket. After the pulse generator was placed, but before the wounds were closed, the clinician programmer was used to measure impedances on the fully implanted system; all impedances on the left-sided electrodes were now found to be elevated beyond the system’s dynamic range (9999+ ohms) consistent with fully open circuits (Table 1, Column B), whereas all right-sided electrode impedances remained within normal limits.

To exclude a malfunction of one of the pulse generator ports or a faulty connection between the lead extension and the port, both extensions were temporarily swapped to the opposite pulse generator port, and the impedances were rechecked. To exclude a faulty lead extension, the connections between the intracranial leads and their lead extensions were swapped, and the impedances were rechecked. Finally, the left lead was detached from its extension and reattached to the external test stimulator, and the impedances were rechecked. In all troubleshooting steps, impedances on all the left-sided electrodes were 9999+, localizing the issue to the lead itself rather than to any proximal connection/component.

Due to the concern for a malfunctioning electrode, we considered replacing it. However, this would have required repeating the application of the head frame, imaging, and planning, which would prolong the anesthesia time and possibly displace the lead from what we had already proven, during the awake monitoring, to be its ideal position. Our multidisciplinary team including a neurosurgeon, neurologist, neuroradiologist, and device company representative reviewed the situation together. We learned from the company representative that, as things were currently configured with this system, impedance testing from the IPG using the patient remote has a higher dynamic range (0-20000 ohms) than does testing using the clinician programmer (0-10000 ohms). Impedances were rechecked using the patient remote and pulse generator and were still elevated but now within the dynamic range, thus allowing us to appreciate a preserved ratio of impedances between circumferential and segmented electrodes (Table 1, Column C).

**Figure 2:**
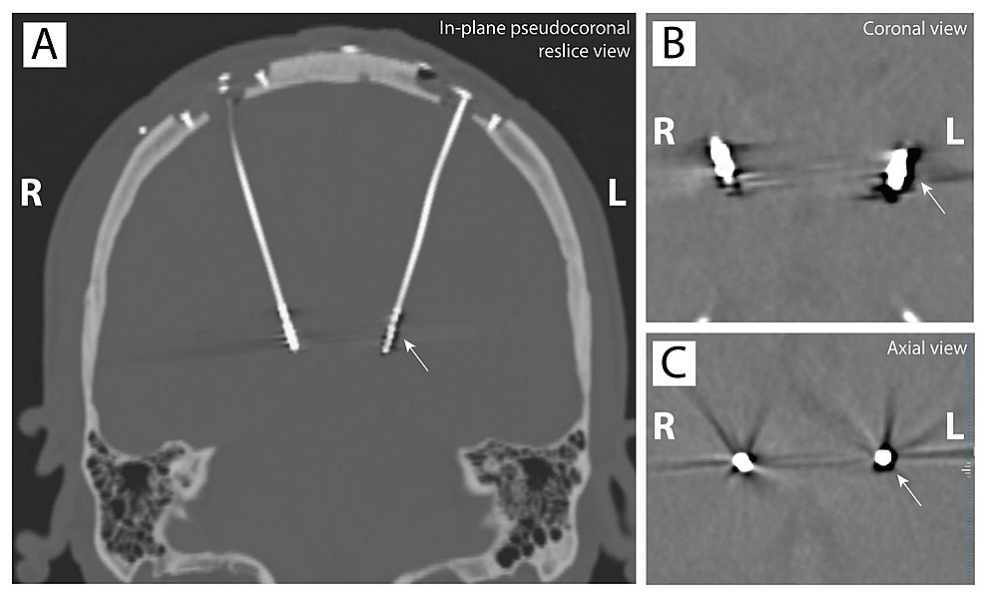
Perioperative CT image from example case 1 (A, B) Coronal (resliced along the plane of the left lead) and (C) axial (resliced orthogonal to the plane of the left lead) non-contrast head CT after ventral intermediate nucleus deep brain stimulation implantation showing air bubble surrounding the left electrode in example case 1.

At this point, we hypothesized that a large bubble of air was surrounding all contacts increasing all impedances proportionally (Figure 1, Panel C). The lead was left in place, and the incisions were closed. We induced a brief Valsalva via the ventilator in an attempt to extrude the air; this resulted in a modest drop in impedances on the circumferential electrodes only (Table 1, Column D). The patient then went for postoperative CT that showed no apparent discontinuity of the connections but revealed a layer of air surrounding the left electrode (Figure 2). Impedances were remeasured after the CT and showed further normalization of the most ventral circumferential electrode impedance (Table 1, Column E), likely consistent with a reduction in the volume of the air bubble (Figure 2, Panel D). The patient went to recovery.

The following day impedances had normalized except for those measured from the contacts of the dorsal-most segmented ring (Table 1, Column F) implying only a small amount of air remained (Figure 2, Panel E). By the first outpatient follow-up visit three weeks after surgery, all impedances were normal (Table 1, Column G).

Case 2

**Figure 3:**
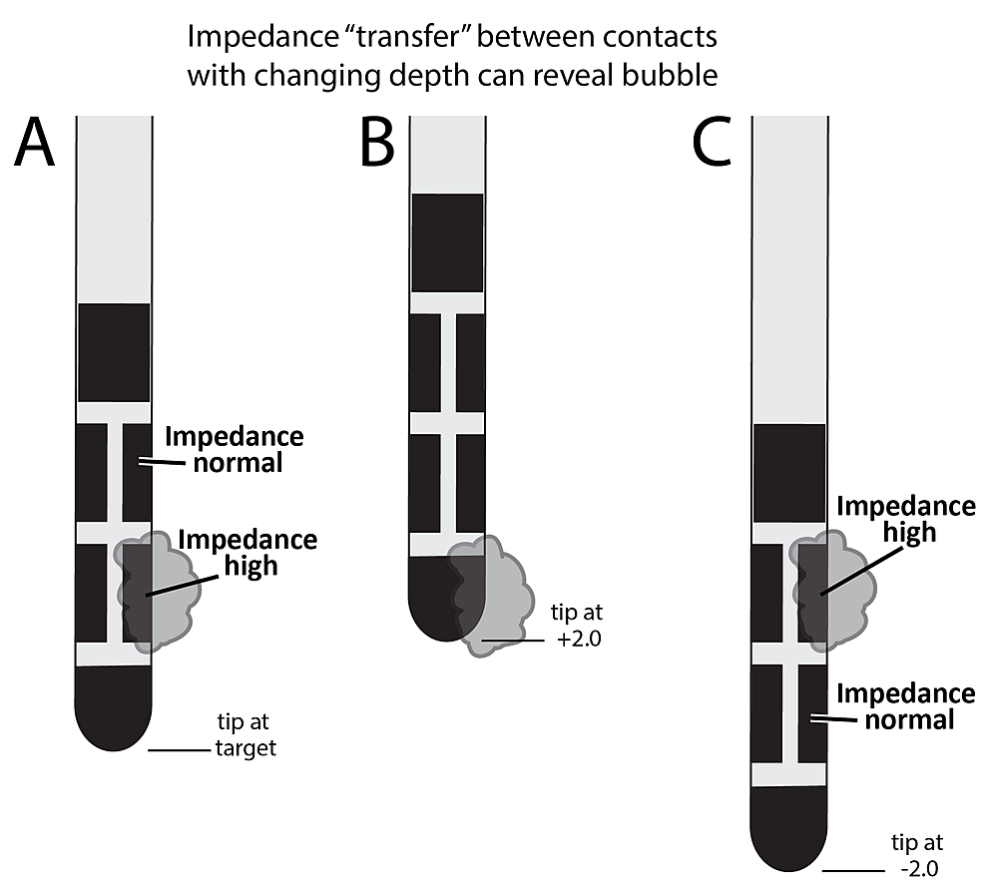
Cartoon illustration describing the second clinical case (A) Air surrounding a single electrode segment with the lead at target, corresponding to elevated impedances in that electrode. (B) As the lead is withdrawn 2 mm above target, the air pocket is fixed, and the impedances normalized. (C) As the lead is inserted 2 mm beyond the target, the air collection remains fixed, corresponding to elevated impedances on the segment immediately superior to the one initially showing abnormal impedances.

A 67-year-old male with medically refractory tremor underwent bilateral Vim thalamic lead insertion using the same approach as described for case 1. When the left lead was placed to target, intraoperative impedances were checked using the external test stimulator, and impedances involving contact 3 (a radially segmented electrode) were found to be elevated beyond the testable range (9999+ ohms) (Table 2, Column A; Figure 3, Panel A). The lead was initially removed, and the tip was placed in a beaker of saline at which point, the impedances were rechecked and found to be normal. When the lead was replaced to target, the impedances were again high on contact 3. We then withdrew the entire lead to 2 mm above target, and the impedances normalized (Table 2, Column B; Figure 3, Panel B). We advanced the lead to 2 mm below target, and the high impedances were now observed on electrode 6, the electrode immediately superior to contact 3 on the lead (Table 2, Column C; Figure 3, Panel C). As this testing confirmed that the high impedance was intrinsic to patient anatomy rather than intrinsic to the lead, we left the lead in place. During the first programming follow-up one month after surgery, all impedances were found to be within normal limits.

**Table 2:** Electrode impedances (in ohms) on the lead illustrated in case 2 See Figure 3.

	Position of Lead Tip		
	A: Target	B: 2 mm above target	C: 2 mm below target
E8	1310	1197	1271
E7	1884	2010	1836
E6	3160	2030	9523
E5	2065	2052	2078
E4	1860	1608	1678
E3	9999+	2243	3167
E2	2506	1893	2067
E1	1295	1291	1245

## Discussion

Testing the impedance to current flow between components of a DBS system can be a valuable tool to ensure that the system is functioning properly both during initial implantation and during subsequent follow-up visits. However, the impedance values taken in isolation may be misleading; therefore, impedance data must be interpreted within their proper context and with a full understanding of what is actually being measured.

For example, it is possible for a DBS system with a complete lead fracture to measure completely normal impedances because electrical current can be transmitted between the exposed wires proximal to the fracture through the surrounding tissue or interstitial fluids [2]. In contrast, impedance values for our cases were elevated to levels consistent with fully open circuits (suggesting lead damage/fracture) even though these systems were, in fact, intact. In our examples, it appears that small pockets of air were interposed at the electrode-tissue interface, impeding current flow. A case similar to our first example, involving elevated intraoperative impedances due to air surrounding Abbott’s infinity directional DBS electrode, has been recently reported by Lyons et al. [3]. Our case differs in that impedances were normal when tested immediately after lead placement but became elevated when rechecked after IPG placement, suggesting a delayed accumulation of air around the lead.

Given the low likelihood that a new DBS lead is deficient at the time of implantation, it has been suggested that confusion regarding spurious initial impedance measurements could be avoided by simply not checking impedances until after the IPG has been implanted [4]. However, in rare cases, we have discovered true lead deficiencies during intraoperative impedance testing, a discovery that has allowed us to avoid repeat stereotactic procedures. Thus, we advocate for intraoperative impedance testing at all possible stages.

Newer DBS systems allow for more fine-tuning of the electrical field by virtue of radially segmented electrodes along the lead [5]. Due to their smaller surface area, these segmented electrodes have higher impedances and are more susceptible to fluctuations in impedance than circumferential electrodes [6]. This has the potential to complicate the assessment of impedances, particularly in the intraoperative setting where air alongside the electrodes is more likely to be present. However, the ratio of impedances between the larger, circumferential electrodes versus the smaller, segmented electrodes can be viewed as a “fingerprint” of the lead which, when preserved in the setting of elevated impedances, points to a cause extrinsic to the lead itself as was the case in our second example.

In order to appreciate these ratios, the impedance values must be within the dynamic range of the impedance measurement. It is not common knowledge, nor necessarily intuitive, that impedance will be measured from the Boston Scientific Gevia™ IPG with a larger dynamic range when using the patient programmer than with the clinician programmer. If out-of-range impedances are seen with this particular setup when using the clinician programmer, they should be rechecked using the patient’s device.

Our second case highlights the relative vulnerability of smaller segmented electrodes to spuriously elevated impedances, and it is particularly interesting to note that in our example, when the lead was positioned such that the air pocket approximated the lowest concentric electrode, there was no apparent effect on the impedances at all despite maximally elevated impedances having been measured when the air pocket abutted a single segment. We have seen elevated impedances isolated to a single segmented electrode in several other cases during intraoperative impedance testing and antidotally found that with a slower descent of the lead to target over 10-15 seconds using a microdrive, the incidence of this phenomenon is less than 10% of leads versus around 25% of leads that are delivered immediately to target.

## Conclusions

Intraoperative impedance testing during DBS surgery is important to ensure that there are no hardware defects in the newly implanted system. Elevated impedances are not uncommon and are more likely to be observed with newer systems employing smaller, radially segmented electrodes. Not all impedances elevations indicate system malfunction, and small pockets of air along the implanted electrodes are likely a common source of elevated impedances and should be distinguishable by a preserved impedance ratio between the smaller radially segmented and larger circumferential electrodes.
